# The Dark Side of Stress Response: Night Temperature Regimes Drive Distinct Abiotic Pathways in Legumes

**DOI:** 10.1111/pce.70276

**Published:** 2025-11-03

**Authors:** Charlotte Häuser, Ashwini Mudke, Kamran Arshad, Hiba Alatrash, Caitlin Dudley, Millicent Smith, Sarah Schiessl

**Affiliations:** ^1^ Department of Genetics of Crop Diversity Justus Liebig University Giessen Germany; ^2^ Sanya Institute of NAU & MARA National Center for Soybean Improvement & State Key Laboratory of Crop Genetics, Germplasm Enhancement and Utilization Nanjing Agricultural University Nanjing China; ^3^ Department of Agricultural Sciences University of Helsinki Finland; ^4^ Centre for Crop Science, Queensland Alliance for Agriculture and Food Innovation The University of Queensland Queensland Australia; ^5^ School of Agriculture and Food Sustainability, Faculty of Science The University of Queensland Queensland Australia

**Keywords:** abiotic stress, high night temperature, legumes, stomatal conductance

## Abstract

Global warming increases night temperatures more strongly than day temperatures. Recent evidence indicates that the effect of night temperature on plant physiology is independent of daytime conditions, suggesting distinct stress tolerance mechanisms. Legume crops, vital to sustainable agriculture, are particularly vulnerable to abiotic stresses, resulting in yield instability. Developing cultivars with round‐the‐clock stress tolerance requires an understanding of the mechanisms for nighttime abiotic stress tolerance. Unfortunately, data on nighttime abiotic stress response remain limited, particularly for legumes. This review examines the current understanding of nighttime versus daytime abiotic stress response in major crop legumes. Our analysis reveals that high night temperatures primarily affect both pod set and grain weight, while high day temperatures predominantly impact pod set, and low night temperatures mainly reduce grain weight. We explore possible underlying mechanisms, including the balance between photosynthesis and respiration rate, nocturnal stomatal conductance, thylakoid membrane structure and composition, as well as the influence of the circadian clock. Notably, we identify a positive trade‐off between warm nights and improved water use efficiency, suggesting promising avenues for breeding climate‐resilient legume varieties for the future.

## Summary statement

Recent evidence suggests distinct stress tolerance mechanisms for high night temperature on plant physiology and yield components in major crop legumes, like altered balance between photosynthesis and respiration, nocturnal stomatal conductance or changes in thylakoid membrane structure.

**Abbreviations**
ABA: abscisic acid
*GA1*
: *gibberellin a1*
*GI*
: *GIGANTEA*
NPQ: non‐photochemical quenching
PSII: photosystem II
QTL: Quantitative Trait Loci
ROS: reactive oxygen species
RSA: root system architecture
*TOC1*
: *TIMING OF CAB EXPRESSION 1*

## 1 Introduction

Legumes represent one of agriculture's most valuable assets, offering exceptional nutritional benefits while contributing significantly to sustainable farming systems. Increasing the cultivation of legumes not only increases agrobiodiversity and provides valuable plant protein but also contributes to sustainable agriculture in various ways. Legumes form symbiotic relationships with nitrogen‐fixing bacteria, reducing the need for fertiliser inputs (Peoples et al. 2009). Many legume species, like faba bean or garden pea, have a high capacity to suppress weeds, decreasing herbicide requirements in farming systems (Verret et al. 2017). They support pollinators through extended flowering periods and high nectar production (Otieno et al. 2020). Furthermore, legumes contain essential micronutrients, particularly calcium, iron and zinc (Campos‐Vega et al. 2010) as well as bioactive compounds with associated health benefits (Dewan et al. 2024). Increasing the proportion of legumes in crop rotations, therefore, serves multiple crucial goals: providing nutritious plant protein and micronutrients for sustainable human diets while enhancing agrobiodiversity, reducing pest and disease pressure, supporting pollinators, and minimising dependence on synthetic N fertilisers and herbicides.

Despite these positive aspects, farmers face significant challenges in legume cultivation, particularly due to low yield stability under abiotic stress, specifically drought and heat. With accelerating climate change, drought and heat waves will further reduce yield if adaptation to abiotic stress is not addressed (IPCC 2019).

Research into the regulation of abiotic stress response in legumes is therefore promising (Araújo et al. 2015). For most legumes, heat stress has the strongest impact on yield during the sensitive phase of early flower development (specifically during gametophyte development) and seed filling (Bishop et al. 2016; Lavania et al. 2015). In addition, legumes are sensitive to drought (Daryanto et al. 2015), which severely impacts yield due to flower abscission (Lavania et al. 2015), reduced outcrossing rates (Bishop et al. 2017), reduced pollen viability and reduced pollen germination (Bishop et al. 2016). Some species like chickpea and lentil suffer more from terminal drought, which causes pod abortion (Farooq et al. 2017). Breeding for adapted cultivars represents a key strategy to overcome this obstacle. However, recent data from non‐legume species indicate that a crop's response to day temperature is independent of its response to night temperature. This could represent a previously unused source of abiotic stress tolerance. Therefore, we like to review the current understanding of night temperature response in legumes to inform and stimulate future research directions.

In this review, we focus on seven economically important legumes: soybean (*Glycine max*), common bean (*Phaseolus vulgaris*), chickpea (*Cicer arietinum*), pea (*Pisum sativum*), mungbean (*Vigna radiata*), lentil (*Lens culinaris*) and faba bean (*Vicia faba*), but also consider minor grain legumes such as pigeon pea (*Cajanus cajan*), groundnut (*Arachis hypogaea*), adzuki bean (*Vigna angularis*) and kidney bean, a variety of common bean (*Phaseolus vulgaris*). Among those, soybean and common bean are most spread in terms of global cultivation area and production, while soybean and faba bean earn the highest yields per hectare (FAOSTAT 2025), have the highest N fixation rates, cover most of their N demand on biological nitrogen fixation and bear the highest protein contents (Peoples et al. 2009) (Table 1). All the same, faba bean is an orphan crop, while soybean ranks among the top five cash crops worldwide. Common bean and chickpea have a considerable role, particularly in the developing world, where they significantly contribute to protein and nutrient supply (Begum et al. 2023; Castro‐Guerrero et al. 2016). Garden pea, mungbean, lentils and faba bean still have limited economic significance, although cultivation areas and interests are rising. Yield instability is particularly high in common bean, faba bean and chickpea, while yield losses due to abiotic stress are especially severe in soybean, faba bean and pea.

**Table 1:** Agricultural significance expressed as global cultivation area [ha], average yield [t/ha] and production [Mt], *N* fixation capacity [kg/ha], percentage of biological nitrogen fixation (BNF) covering the plant's *N* demand [%] and protein content of seeds [%] for seven grain legumes selected for this review.

Crop	Global cultivation area [ha]	Average yield [t/ha]	Production[Mt]	*N* fixation capacity [kg/ha]	BNF covering *N* demand [%]	Protein content [%]
Soybean	125	2.711	371.2	119	68	36
Common bean	37.8	0.755	28.5	48	39	20
Chickpea	14.8	1.171	16.5	51	58	19
Garden pea	7.4	1.857	13.8	86	65	25–30
Mungbean	6	NA	NA	58–109	NA	24
Lentils	5.8	1.245	70.6	82	65	25
Faba bean	2.7	2.183	6.1	129	75	29

*Note:* % = percent data for cultivation area, yield and production are sourced from FAOSTAT (2025). Data for *N* fixation and BNF are sourced from Peoples et al. (2009).

Abbreviations: ha, hectare; kg/ha, kilograms per hectare; Mt, million tons; t/ha, tons per hectare.

## 2 Legumes Are Distinct in Abiotic Stress Response

Abiotic stress responses are complex, and adaptation strategies vary between species. Here, we summarise the most important legume‐specific traits related to abiotic stress. Comprehensive insights into abiotic stress responses in model legumes, as well as grain and forage legumes, have been extensively reviewed in the literature (Hasanuzzaman et al. 2020).

### 2.1 Indeterminate Growth Is Beneficial for Abiotic Stress Resilience in Legumes

Indeterminate growth refers to a growth pattern where the apical meristem remains active, allowing continuous growth and development of new tissues throughout the plant's life. This contrasts with determinate growth, where growth ceases after a certain point, often after flowering. Indeterminate growth is common in wild relatives of legumes as well as in most grain legumes, although there is genotypic variation. This growth habit is characterised by persistent meristem activity above‐ and below‐ground. This includes persistent meristems in nodules, which allow for ongoing development and nitrogen fixation. Under fluctuating environmental conditions, indeterminate growth can be advantageous as it allows continuous development. For instance, even after full flower abortion, plants can form more flowers after the stress event, reducing risks for farmers. Indeterminate growth can support sustained nitrogen fixation, which is crucial for stress resilience in legumes (Matamoros and Becana 2021).

### 2.2 Root System Architecture (RSA) and Nodule Formation Enhance Abiotic Stress Tolerance of Legumes

Crop performance is closely related to the development of the root system, which is strongly affected by different stress environments in the soil. Legumes have great genetic diversity in RSA, providing an advantage in adaptation (Belachew et al. 2018; Ye et al. 2018). As dicots, legumes typically develop deep, fibrous taproot systems, enabling efficient water and nutrient uptake and improving their ability to handle abiotic stresses like drought and nutrient deficiency (Khan et al. 2010; Ribeiro et al. 2019). To the best of our knowledge, research into the impact of temperature on RSA in grain legumes has not been carried out.

Legume roots differ from many other crop root systems in their ability to fix atmospheric N_2_ (Khan et al. 2010; Ribeiro et al. 2019), an important feature in reducing the demand for N fertilisation, but also with respect to biological nitrification inhibition (BNI). Moreover, the size and activity of nodules are correlated with drought tolerance in legumes (Z. Wang et al. 2024). However, nodule formation, essential for biological N_2_ fixation in legumes, is substantially influenced by abiotic stress. For example, water deficit conditions negatively affect *Rhizobium* populations in the soil, impairing rhizobium‐legume symbiosis (Zhang et al. 2024). The onset of nodules in grain legumes is generally delayed by soil temperatures outside the optimal range, particularly low soil temperatures (Lira Junior et al. 2005).

## 3 Night Temperatures Have a Strong Influence on Stress Response

There is growing evidence that the effect of high or low temperatures on physiology and yield depends on timing. A meta‐analysis by Jing et al. (2016) highlighted that the effects of either high or low night temperature are species‐specific and cannot be generalised (Jing et al. 2016). For example, in C3 plants, high night temperatures stimulated, decreased or unchanged the net CO_2_ assimilation rate and biomass accumulation, yet consistently reduced yield (Jing et al. 2016).

While some authors attribute the yield reduction to a reduction in biomass (Lin et al. 2021), the above‐mentioned meta‐analysis concluded this relationship holds true only for low night temperatures, as high night temperatures, by contrast, led to suboptimal resource allocation to reproductive organs (Jing et al. 2016). As legumes tend to have particularly low values for harvest index, the latter appears more likely.

Unfortunately, climate change is causing night temperatures to rise more quickly than day temperatures, with high night temperatures persisting longer (Davy et al. 2017). Notably, research in cereals has shown that some genotypes classified as heat‐tolerant exhibited stronger yield decreases in response to high night temperatures. This suggests that day and night heat tolerance mechanisms may be uncoupled (McAusland et al. 2023), potentially threatening currently tolerant varieties as night temperatures continue to rise. From a breeding perspective, however, this presents an opportunity to combine independent tolerance strategies to develop cultivars with all‐day tolerance.

### 3.1 What Is Optimal Temperature?

The temperature considered optimal during the day and during the night varies strongly between different legumes (Table 2). Some species, like faba bean and mungbean, show pronounced drops of 16°C–20°C in optimal temperature between day and night, while data for pea suggest a much lower gap in optimal day and night temperatures, with 10°C–15°C gaps being frequent. Research in faba bean indicates that the optimum night temperature decreases as plants mature (McDonald and Paulsen 1997), suggesting that different developmental stages may require different optimal temperatures. Data from mungbean imply that the optimal night temperature might depend on the corresponding day temperature, as yield increased with night temperature under non‐stress day conditions, while it decreased under daytime heat stress (Williams et al. 2022). Rather than discussing optimal day and night conditions separately, it might be more appropriate to consider optimal temperature regimes. Irrespective of the developmental stage and species, plants generally tolerate much higher temperatures during the day than during the night.

**Table 2:** Optimal day and night temperatures for grain legumes.

Crop	Optimal day temperature	Optimal night temperature	References
Soybean	< 35°C	20°C–27°C	Ding et al. (2023); Lin et al. (2021)
Common bean	29°C–34°C	20°C	Albertine and Manning (2009)
Chickpea	< 35°C	15°C–20°C	Gaur et al. (2014); Kumari et al. (2020)
Garden pea	21°C–25°C	13°C–21°C	Serova et al. (2023); Stanfield et al. (1966)
Mungbean	30°C	20°C	Chauhan and Williams (2018); Rachaputi et al. (2019); Williams et al. (2022)
Lentil	28°C–30°C	20°C–23°C	Bhandari et al. (2016); Sehgal et al. (2019)
Faba bean	< 26°C	10°C	Bishop et al. (2016); Bishop et al. (2016); Said et al. (1967)
Pigeon pea	24°C–32°C	16°C–24°C	McPHERSON et al. (1985)
Groundnut	30°C–35°C	22°C–26°C	Kakani et al. (2002); Massawe et al. (2003); P. V. Prasad et al. (2000)
Adzuki bean	17°C–20°C	7°C–10°C	Xiang et al. (2024)
Kidney bean	26°C–40°C	15°C–30°C	Jifon and Wolfe (2005); P. V. V. Prasad et al. (2002)

### 3.2 How Is High Night Temperature Response Different From Daytime Heat Response?

Heat stress negatively affects legume yield depending on intensity and duration, with varying sensitivities among species (Table 2). For example, chickpea yields can decrease by 10%–15% for every 1°C increase above the optimal day temperature (Upadhyaya et al. 2011). In pea, a 1°C rise in mean temperature during flowering can reduce production by 0.6–0.8 t/ha (Ridge and Pye 1985; Lamichaney et al. 2021).

The reproductive stage is consistently the most vulnerable to heat stress across all legumes during both night and day (Farooq et al. 2017). We have a fairly good understanding of daytime heat stress response in legumes (please refer to excellent reviews elsewhere [Farooq et al. 2017]) with several identified physiological and molecular tolerance mechanisms, including expression of heat‐shock proteins, altered membrane composition, antioxidative processes, osmotic adjustment and transpiration cooling. Daytime heat stress tolerance is undoubtedly crucial for yield stability, specifically during reproduction (Farooq et al. 2017). However, data from cereals indicates that night and day temperature stress responses operate independently, with some of the most heat‐tolerant genotypes showing the lowest tolerance to high night temperatures (McAusland et al. 2023). This underscores the need to evaluate which traits are specifically affected by either day or night temperature in legumes and to determine what functional differences exist.

Only a few studies have isolated the influence of night temperature by comparing different night temperatures while maintaining constant day temperature in legumes. Nevertheless, such data are available for soybean (Djanaguiraman et al. 2013; Gibson and Mullen 1996a; Lin et al. 2021; Shu 2021; Yang et al. 2023; Zheng et al. 2002), faba bean (Said et al. 1967), pea (Stanfield et al. 1966), common bean (Cayetano‐Marcial et al. 2021) and mungbean (Williams et al. 2022). An additional relevant study available for pea focused on the effect on nodules, but not on yield (Serova et al. 2023), while another study in common bean was focused on biomass and ozone injury, but not yield (Albertine and Manning 2009). Genetic diversity in these studies is typically limited, with the maximum genetic diversity studied being three and nine genotypes (Lin et al. 2021; Shu 2021). Despite these limitations, these studies have revealed substantial variation in high night temperature response, indicating significant potential for improvement through breeding.

#### 3.2.1 Yield, Yield Components and Seed Quality

High night temperatures generally result in negative effects on yield or total seed weight per plant in most legumes studied, including soybean (Djanaguiraman et al. 2013; Lin et al. 2021; Shu 2021; Yang et al. 2023), pea (Stanfield et al. 1966) and faba bean (Said et al. 1967). However, two soybean studies (Gibson and Mullen 1996a; Zheng et al. 2002) and one common bean study (Cayetano‐Marcial et al. 2021) did not observe reduced yield. This variability may be explained by a finding in mungbean where higher night temperatures increased yield under optimal day temperatures, but decreased yield when day temperatures were too high (Williams et al. 2022).

The magnitude of these effects varies considerably. Gibson and Mullen (1996a) found that high night temperatures had a smaller effect on soybean yield components compared to high day temperatures, while Djanaguiraman et al. (2013) observed the opposite. High night temperatures typically reduced pod set or pod number (Djanaguiraman et al. 2013; Lin et al. 2021; Said et al. 1967; Yang et al. 2023). Also, seed or pod weight has been decreased (Gibson and Mullen 1996a; Said et al. 1967; Shu 2021; Yang et al. 2023; Zheng et al. 2002) most likely as a consequence of smaller seed size (Yang et al. 2023).

Effects on total seed number show mixed results: one study reported increased seeds per plant (Zheng et al. 2002) while three studies reported fewer seeds per plant (Said et al. 1967; Yang et al. 2023) or reduced seeds per pod (Williams et al. 2022). Lin et al. (2021) reported higher node number in soybean, while harvest index remained unchanged in soybean and common bean (Cayetano‐Marcial et al. 2021; Lin et al. 2021; Shu 2021). Interestingly, high night temperatures appear to influence pod distribution throughout the plant by stimulating increased pod set in secondary and tertiary racemes in soybean (Gibson and Mullen 1996b; Zheng et al. 2002). A similar effect was observed with different night/day regimes in faba bean, where pod set shifted from lower nodes towards higher nodes in faba bean (Bishop et al. 2016), possibly indicating better resource allocation to flowers throughout the plant.

**Figure 1:**
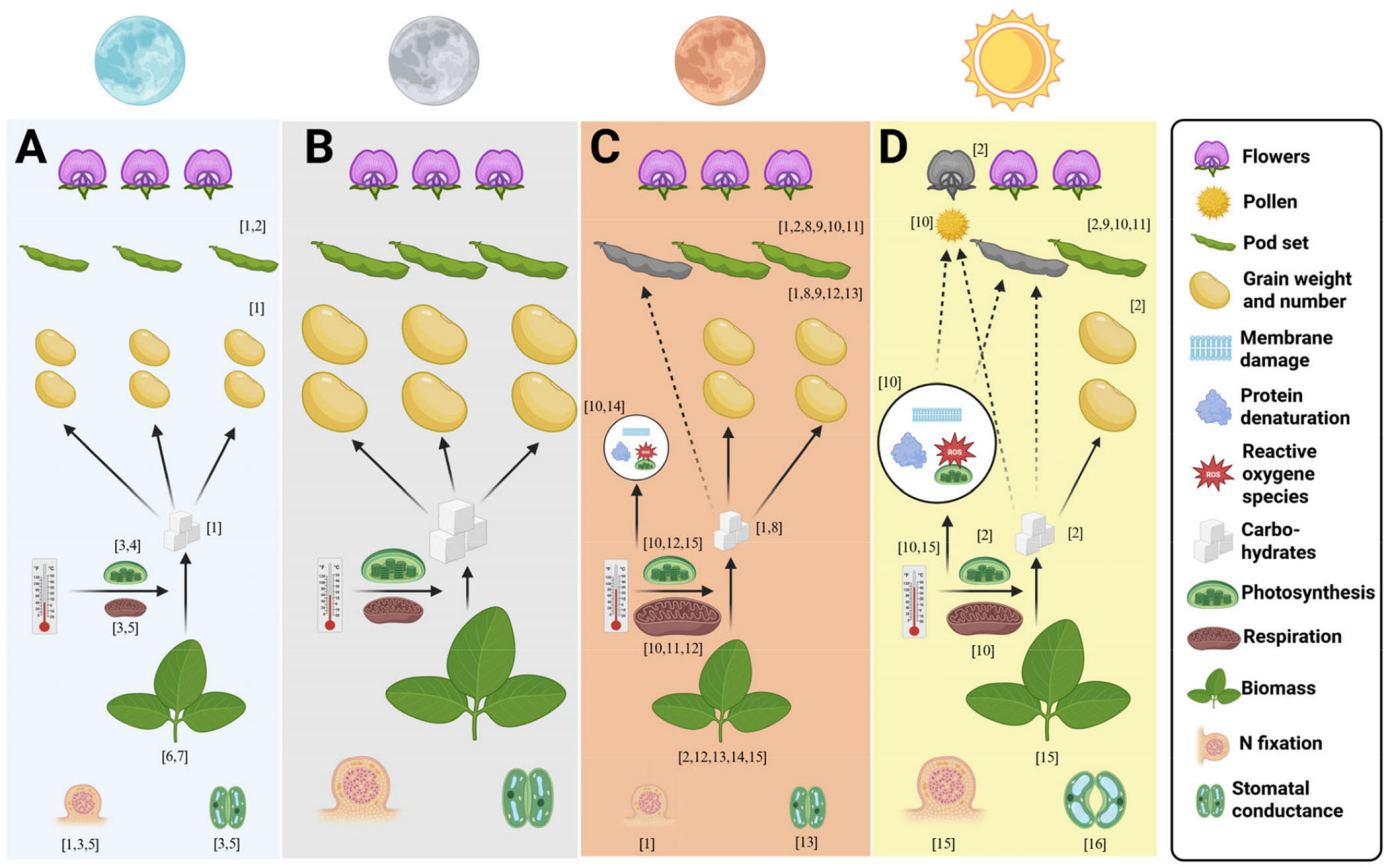
Influence of (A) low night temperatures, (B) ambient night temperatures, (C) high night temperatures and (D) high day temperatures on grain legumes. In contrast to day conditions, where lack of carbohydrates and cellular stress seem to accelerate floral abortion (indicated as grey flower), night temperature generally seems to have no influence on floral abortion. Under high night temperatures, similar to day conditions, pod set is affected (indicated as grey pod), most likely linked to a lack of available carbohydrates due to an imbalance between photosynthesis and respiration. While high day temperatures seem to have no effect on biomass, both low and high night temperatures reduce it, although for different reasons. References: [1] (Said et al. 1967), [2] (Farooq et al. 2017), [3] (Van Heerden and Krüger 2004), [4] (Polishchuk et al. 2020), [5] (Van Heerden et al. 2003), [6] (Stanfield et al. 1966), [7] (Ormrod 1979), [8] (Yang et al. 2023), [9] (Williams et al. 2022), [10] (Djanaguiraman et al. 2013), [11] (Lin et al. 2021), [12] (Shu 2021) [13] (Cayetano‐Marcial et al. 2021), [14] (Albertine and Manning 2009), [15] (McDonald and Paulsen 1997), [16] (Sehgal et al. 2019). Created in BioRender.

Daytime heat stress during flowering and pod set increases flower and pod abortion in various legumes, including soybean (Soba et al. 2022), faba bean (Bishop et al. 2016; Bishop et al. 2016), mungbean (Khattak et al. 2009) and chickpea (Devasirvatham et al. 2015), primarily reducing yield due to reduced seed number (Bishop et al. 2016). For faba bean, pollinators like bumblebees can mitigate these negative effects by improving pod allocation across the plant towards more productive nodes, resulting in increased seed set (Bishop et al. 2016). A summary of our findings regarding different temperature regimes on yield components can be found in Figure 1.

Research on soybean suggests that diurnal temperature fluctuations have a stronger influence on protein and oil content than day temperatures alone (Song et al. 2016). This implies that night temperature may be a stronger driver of seed composition than day temperature.

Studies show contrasting effects across different legume species. In soybean, both oil and protein content increased under high night temperatures (Yang et al. 2023), however research in faba bean demonstrated lower seed nitrogen content under high night temperatures (Said et al. 1967), suggesting species‐specific responses to nighttime thermal conditions.

#### 3.2.2 Reproductive Traits

While high day temperatures are known to accelerate flowering time in legumes, high night temperatures have contrasting effects, actually resulting in delayed flowering in faba bean (Said et al. 1967). Further data from other legume species are required to disentangle day and night temperature effects on phenology more broadly.

The impact on reproductive function also appears to vary. In soybeans, high night temperatures reduced both pollen viability and pollen germination (Djanaguiraman et al. 2013), potentially compromising fertilisation. Conversely, another study observed increased flower production in soybean under elevated night temperatures (Zheng et al. 2002), suggesting complex responses in reproductive development that may vary by genotype or experimental conditions.

#### 3.2.3 Photosynthesis and Respiration

Research on photosynthetic responses to high temperatures reveals mixed results, indicating complex interactions. Chlorophyll content responses vary significantly across studies, with some reporting decreases (Djanaguiraman et al. 2013; McDonald and Paulsen 1997; Shu 2021) associated with lower photosystem II (PSII) quantum yield, lower electron transport rate (Djanaguiraman et al. 2013) and decreased photosynthetic rates (Djanaguiraman et al. 2013; Shu 2021). Conversely, other studies report increased chlorophyll content associated with higher photosynthetic rates and CO_2_ fixation (Shu 2021; Yang et al. 2023). Results from McDonald and Paulsen (1997) and Lin et al. (2021) were not conclusive. These contradicting findings may be explained by temporal dynamics in plant responses. Yang et al. (2023) found that high night temperatures initially increase photosynthesis rates, but after prolonged exposure, photosynthesis returned to control levels—although this pattern was genotype‐dependent.

In contrast to the variable photosynthetic responses, night respiration rates consistently increase under high night temperatures (Djanaguiraman et al. 2013; Lin et al. 2021) with greater increases than those observed under daytime heat stress (Djanaguiraman et al. 2013). Lin et al. (2021) quantified this increase at approximately 9.6% per 1°C night temperature rise, although Shu (2021) found these effects to be time‐ and intensity‐dependent.

#### 3.2.4 Carbohydrate Availability

High night temperatures alter carbohydrate distribution and metabolism in legumes. In both soybean and faba bean, seed and stem sucrose decreased significantly under higher night temperatures (Said et al. 1967; Yang et al. 2023). Faba bean also shows reduced seed glucose content (Said et al. 1967). Leaf sucrose in soybean initially increased, but later returned to normal levels (Yang et al. 2023).

Starch follows a similar pattern, with reduced concentrations in seeds and stems, but relatively stable levels in leaves (Said et al. 1967; Yang et al. 2023). These carbohydrate alterations align with the observed increases in oil and protein content (Song et al. 2016; Yang et al. 2023) and with the increased respiration rate under high night temperatures (Djanaguiraman et al. 2013; Lin et al. 2021). This suggests that increased nighttime respiration consumes carbohydrate reserves that would otherwise contribute to seed filling.

#### 3.2.5 Biomass and Plant Architecture

High night temperatures produce varied effects on dry matter accumulation in legume species. In soybean, dry matter consistently decreases under high night temperatures (Lin et al. 2021; Shu 2021; Yang et al. 2023), with similar reductions observed in common bean and faba bean (McDonald and Paulsen 1997). In mungbean, the response depends on daytime temperature: higher night temperatures increase dry matter content when day temperatures are optimal, but decrease dry matter under daytime heat stress (Williams et al. 2022). Soybean responses are similarly modulated by light intensity during the day (Shu 2021). Research on pea and common bean has produced conflicting results. Stanfield et al. (1966) found that high night temperatures increased dry matter, while high day temperatures decreased it, while McDonald and Paulsen (1997) reported the exact opposite pattern, and no change was noted by Albertine and Manning (2009) and Cayetano‐Marcial et al. (2021).

Growth rate increases under high day temperatures in common bean and faba bean (McDonald and Paulsen 1997; Stanfield et al. 1966), but responses to high night temperatures are inconsistent for pea (Albertine and Manning 2009; McDonald and Paulsen 1997).

Leaf area remains either unchanged (McDonald and Paulsen 1997; Shu 2021) or increases (Albertine and Manning 2009) under high night temperatures. Leaf number increased in common bean and pea, but not in faba bean (McDonald and Paulsen 1997), while node number increased in soybean (Shu 2021).

Plant height responses vary by species, with high night temperatures increasing height in common bean (Albertine and Manning 2009) and soybean (Shu 2021) but reducing height in faba bean (Said et al. 1967) and pea (Stanfield et al. 1966).

Data on root architecture under high night temperatures are limited. A preliminary assessment of shoot‐to‐root ratio in soybean found decreases under high night temperatures, suggesting increased resource allocation belowground (Lin et al. 2021). In addition, prolonged daytime heat leads to a loss of nodules in pea, which can be partially avoided by optimal night temperatures (Serova et al. 2023). Moreover, nodulation depends on synchronisation of the circadian clock between the host and symbiont (Rowson et al. 2024), and optimal night temperatures are crucial for recovery of harmed nodules damaged by daytime stress (Serova et al. 2023).

#### 3.2.6 Oxidative Stress

High night temperatures negatively impact cellular integrity in legumes, causing greater membrane damage compared to ambient night temperatures in both common bean and soybean (Albertine and Manning 2009; Djanaguiraman et al. 2013). However, daytime heat stress typically produces more severe membrane damage than nighttime heat (Djanaguiraman et al. 2013), suggesting different mechanisms or intensities of stress response between day and night conditions.

Non‐photochemical quenching (NPQ), a key photoprotective mechanism, showed varied responses to high night temperatures. Djanaguiraman et al. (2013) found that NPQ increased under high night temperatures in soybean even more so than under daytime heat stress. This suggests nighttime thermal conditions may influence subsequent daytime photosynthetic efficiency through persistent effects on the photosynthetic apparatus. However, these findings are not conclusive or may be genotype dependent, as Lin et al. (2021) observed no significant effect on NPQ under similar conditions.

### 3.3 How Is Low Night Temperature Response Different From Daytime Cold Response?

Cold stress in plants generally encompasses two temperature ranges: frost stress (temperatures below 0°C) and chilling stress (temperatures above 0°C, but too low to support growth). Lentil, faba bean, pea and chickpea are cold‐adapted legumes with varying levels of frost tolerance (Croser et al. 2003; Henriquez et al. 2017; Ridge and Pye 1985; Shafiq et al. 2012). Winter faba bean cultivars demonstrate remarkable frost tolerance, with some cultivars surviving extended periods (10+ days) below −10°C (Carrillo‐Perdomo et al. 2022). Chickpea generally survives minimum temperatures of –8°C, though some genotypes can tolerate temperatures as low as –12°C after emergence (Croser et al. 2003). Quantitative trait loci associated with frost tolerance have been identified for both faba bean (Ali et al. 2016; Arbaoui et al. 2008; Carrillo‐Perdomo et al. 2022) and chickpea (Kalve et al. 2023), indicating potential targets for breeding programs.

The range of temperatures experienced as chilling stress is large. Like drought and heat stress, cold stress impacts vary with developmental stage and duration, with flowering being the most critical, although seedlings are often more likely to be hit by cold temperatures. Chickpea seedlings are commonly exposed to low temperatures (3°C–13°C) that can decrease seedling vigour and ultimately reduce germination rates (Bakht et al. 2006; Zeitelhofer et al. 2022).

During flowering, daytime chilling stress can cause substantial yield losses, primarily due to abortion of buds, flowers, and recently set pods, as well as pod death and a reduction in seed size (Ridge and Pye 1985; Shafiq et al. 2012). These yield reductions are associated with stunted plant growth, reduced biomass production, reduced levels of carbohydrates in the anthers and pollen (Croser et al. 2003; Rani et al. 2021), a reduction in the maximum quantum efficiency of PSII (Fv/Fm), a decrease in net photosynthetic rate (PN), and a reduction in chlorophyll content (Zeitelhofer et al. 2022).

Considerable knowledge already exists on daytime cold tolerance variation in soybean and chickpea, with several QTL and candidate genes identified for both chilling stress (Mugabe et al. 2019; Sun et al. 2020; Zhou et al. 2008) and frost stress (Kalve et al. 2023).

In contrast, data for low night temperature effects are scarce and often decades old. To the best of our knowledge, no data on nighttime frost is available. Most suitable studies on nighttime chilling stress come from soybean (Seddigh et al. 1988, 1989; Van Heerden and Krüger 2004; Van Heerden et al. 2003), pea (Polishchuk et al. 2020; Stanfield et al. 1966; Stavang et al. 2007), faba bean (Ellis et al. 1988; Said et al. 1967), lentil (Summerfield et al. 1985) and common bean (Ormrod 1979).

#### 3.3.1 Yield, Yield Components and Seed Quality

Only two studies from faba bean and pea included yield‐related traits under low night temperature conditions (Said et al. 1967; Stanfield et al. 1966). Both reported reduced yield under low night temperatures, although it remains unclear if the effect is weaker or stronger than the response to low day temperatures. Pod and seed number remain unchanged under low night temperature (Said et al. 1967; Stanfield et al. 1966), but pod weight per plant decreased (Said et al. 1967). Seed nitrogen content also decreased under these conditions (Said et al. 1967). The results are summarised in Figure 1.

#### 3.3.2 Reproductive Traits

Flowering time was consistently delayed under low night temperatures across four legume species (faba bean [Said et al. 1967], soybean [Seddigh et al. 1989], lentil [Summerfield et al. 1985] and pea [Stanfield et al. 1966]). Results on maturation time are however mixed, possibly influenced by photoperiod interactions (Seddigh et al. 1989; Stanfield et al. 1966). However, the influence on maturation was clearly smaller than on flowering time. We could not find any data on pollen viability or germination under low night temperatures.

#### 3.3.3 Photosynthesis and Carbohydrate Availability

CO_2_ fixation rate, electron transport rate, as well as PSII activity all decreased in soybean and pea subjected to low night temperatures compared to controls (Polishchuk et al. 2020; Van Heerden et al. 2003; Van Heerden and Krüger 2004). Reduced photosynthetic capacity was also associated with lower stomatal conductance in soybean (Van Heerden et al. 2003; Van Heerden and Krüger 2004). Van Heerden and Krüger (2004) reported that the CO_2_ fixation rate and PSII activity recovered after low night temperatures ended.

We could not find data on respiration rate under low night temperatures. Said et al. (1967) reported that faba bean seeds under low night temperature had lower glucose, sucrose and polysaccharide content. Nitrogen fixation in soybean was also reduced upon low night temperatures and did not recover after normal temperature conditions resumed (Van Heerden et al. 2003; Van Heerden and Krüger 2004).

#### 3.3.4 Biomass and Plant Architecture

Both dry and fresh matter decreased under low night temperatures in pea and common bean (Ormrod 1979; Stanfield et al. 1966). This aligns with findings of reduced plant height in faba bean, common bean and pea (Ormrod 1979; Polishchuk et al. 2020; Said et al. 1967), reduced leaf area in pea (Polishchuk et al. 2020) and lower growth rate in pea (Stanfield et al. 1966; Stavang et al. 2007). Interestingly, Stavang et al. (2007) found that gibberellin A1 (GA1) concentration increases in response to temperature drops during the day but not during the night, suggesting distinct regulatory mechanisms.

### 3.4 Why Are Legumes More Heat‐Tolerant and Less Cold‐Tolerant During the Day?

The differential temperature tolerance between day and night is not trivial. While stress conditions fundamentally differ at night due to a drop in temperature and the absence of light—changes anticipated by the circadian clock—the decreasing difference between day and night temperatures due to climate change necessitates understanding the mechanisms behind these differential responses.

**Figure 2:**
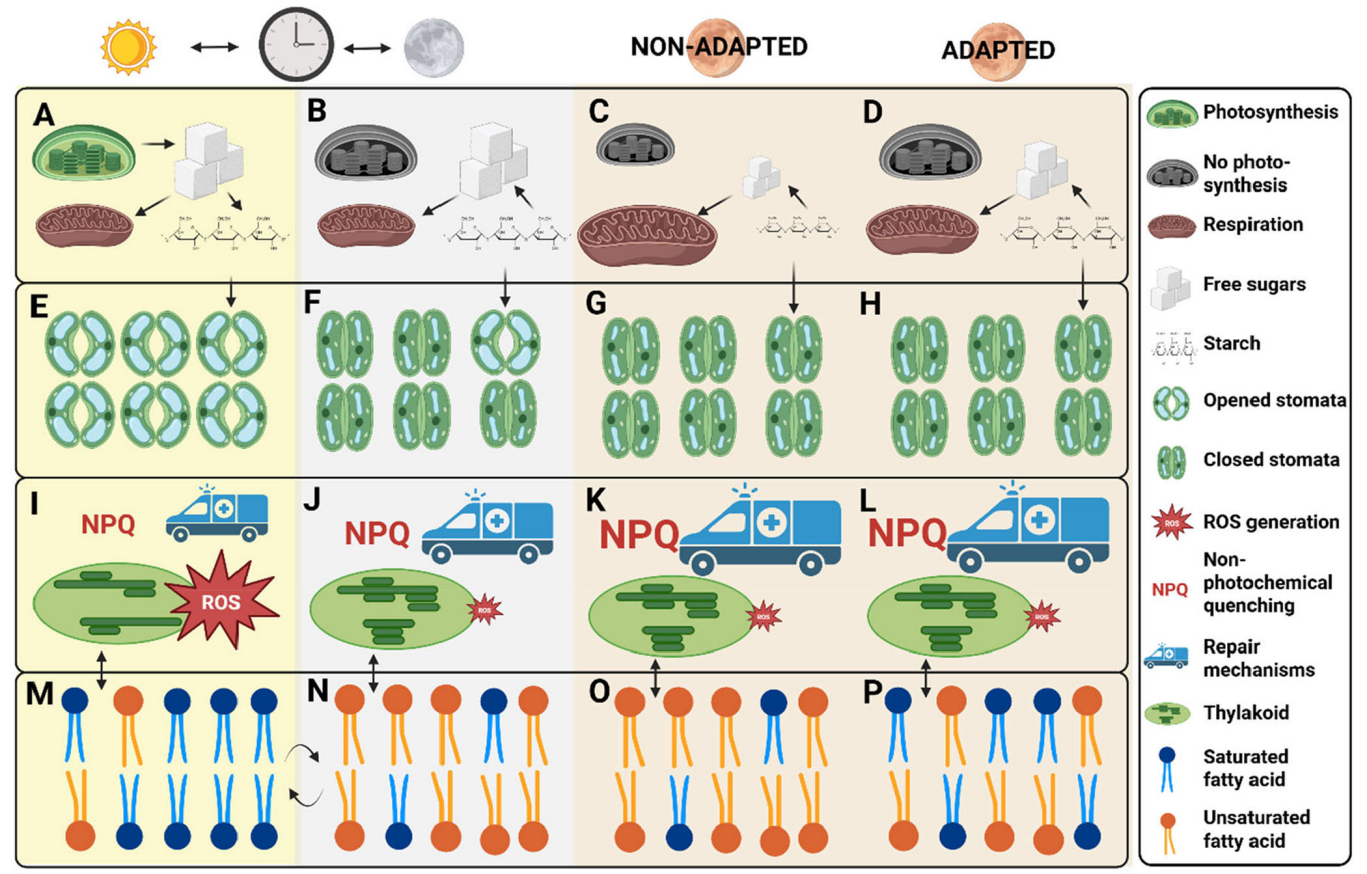
Summary of possible mechanisms for differential response to high night temperature. Yellow background (A, E, I, M): process under ambient day conditions. Grey background (B, F, J, N): process under ambient night conditions. Orange background, non‐adapted (C, G, K, O): process under high night temperature in a non‐adapted legume, orange background, adapted (D, H, L, P): process under high night temperature in an adapted legume. (A–D) Depiction of photosynthesis—respiration balance. In non‐adapted legumes, decreased photosynthesis and/or increased respiration rate decrease the availability of starch and free sugars, while adapted legumes optimise this balance and suffer less. (E–H) Depiction of stomatal conductance. Under ambient night conditions, there is residual stomatal conductance, while in high night temperatures, residual transpiration is abolished, possibly in connection with lower starch availability and water use efficiency is increased. Adapted legumes also keep this behaviour in higher starch concentrations. (I–L) Depiction of ROS generation and repair. In contrast to day conditions, ROS generation during the night is very low. However, during the night, thylakoids change their structure, which is associated with higher rates of NPQ. This process is accelerated in high night temperatures, which is likely to be beneficial. (M–P) Depiction of membrane composition. During the night, the degree of saturated fatty acids decreases to optimise membrane fluidity at expected temperatures. Adapted legumes should decrease this content less to reach optimal membrane structure. This could have an influence on thylakoid stacking. Created in BioRender. [Color figure can be viewed at wileyonlinelibrary.com]

During the night, plants mitigate the negative impacts of heat on membrane and protein stability, conserve energy by downregulating photorespiration, and save water by regulating stomatal conductance. However, while daytime stomatal opening depends on water availability and CO_2_ concentration (K. S. L. Johansson et al. 2020; Vicente‐Serrano et al. 2022), nighttime stomatal opening depends on the previous day's photosynthetic activity (Easlon and Richards 2009). This example highlights the need for a better understanding of interactions between day‐dependent and night‐dependent stress responses to identify optimal plant traits. A summary of our findings is presented in Figure 2.

#### 3.4.1 Membrane Composition

Rikin et al. (1993) found in cotton that heat resistance increases during the day after the onset of light, with a second peak in the middle of the night, linked to a diurnal oscillation in membrane lipid composition. Under light conditions, the degree of more saturated membrane lipids increases, enhancing membrane stability under heat stress (Nakamura 2018). Conversely, saturation decreases at night, improving membrane stability under chilling conditions (Rikin et al. 1993). Although data remain limited and no regulatory mechanisms have been fully characterised so far, this represents a potential major mechanism underlying differential heat tolerance during day and night. For high night temperature tolerance, legume varieties with higher membrane saturation levels might offer advantages for crop improvement.

#### 3.4.2 Growth Rate and Carbohydrate Allocation

Higher temperatures accelerate biochemical processes. When biological structures remain uncompromised by heat, higher growth rates are expected at higher temperatures and decreased rates at lower temperatures, irrespective of daytime—a pattern observed across plant types (Jing et al. 2016). This meta‐analysis concluded that low night temperatures primarily reduce yield due to decreasing biomass, while high night temperatures reduce yield through suboptimal resource allocation to reproductive organs (Jing et al. 2016).

For legumes, photosynthetic processes decrease under low night temperatures (Polishchuk et al. 2020; Van Heerden et al. 2003; Van Heerden and Krüger 2004), with consistent reductions in biomass and growth rate (Ormrod 1979; Polishchuk et al. 2020; Said et al. 1967; Stanfield et al. 1966; Stavang et al. 2007). Consequently, seed weight, rather than seed number, is affected, indicating that the source‐sink ratio predominantly influences yield reduction (Said et al. 1967; Stanfield et al. 1966). Stavang et al. (2007) found that downregulation of growth rate during the day is actively regulated by phytohormones, while nighttime reduction seems more passive. This mirrors the expected negative impact of cold: during the night (common, expected) versus day (unexpected, indicates unfavourable conditions). In conclusion, low night temperatures primarily affect legume yield by a slowdown in metabolism and an unfavourable source‐sink ratio, resulting in smaller seeds.

High night temperature effects present a more complex picture. Respiration rates increase under high night temperatures (Djanaguiraman et al. 2013; Lin et al. 2021; Shu 2021), while increases in daytime photosynthesis are only transient, leading to long‐term carbohydrate deficits (Shu 2021; Yang et al. 2023). Elevated respiration has been associated with energy‐demanding repair processes (Jumrani and Bhatia 2014; Shu 2021), suggesting repair processes are more active at night.

While higher biomass under high night temperatures often occurs under certain conditions like absence of stress during the day (Stanfield et al. 1966; Williams et al. 2022), other studies show unchanged (Albertine and Manning 2009; Cayetano‐Marcial et al. 2021) or reduced growth (Lin et al. 2021; McDonald and Paulsen 1997; Shu 2021; Yang et al. 2023). Beyond certain temperature thresholds, it appears that legumes cannot translate higher growth rates into yield. Reduced growth was associated with lighter (but not smaller) leaves (McDonald and Paulsen 1997) and a lack of carbohydrates, mainly in stems and seeds (Said et al. 1967; Yang et al. 2023). Unlike with low night temperatures, both pod number and seed weight are negatively affected, while harvest index remains unchanged—indicating that source capacity remains the limiting factor under high night temperatures. High night temperatures therefore reduce available carbohydrates (Said et al. 1967; Yang et al. 2023) reducing pod set (Djanaguiraman et al. 2013; Lin et al. 2021; Said et al. 1967; Yang et al. 2023), seed weight (Shu 2021; Yang et al. 2023; Zheng et al. 2002) and yield (Djanaguiraman et al. 2013; Lin et al. 2021; Said et al. 1967; Yang et al. 2023) due to an imbalance between photosynthesis and respiration (Djanaguiraman et al. 2013; Lin et al. 2021; Shu 2021), supporting the hypothesis from Jing et al. (2016).

#### 3.4.3 Transpiration

Plants generally close stomata at night, reducing water loss, but also decreasing cooling capacity. The enhanced daytime cooling partly explains why plants tolerate higher temperatures during the day. However, partial nighttime stomatal opening has been observed in many species, resulting in 5%–15% of daytime water loss as summarised by Easlon and Richards (2009). This effect depends on the previous day's photosynthetic rate and is mediated by starch metabolism, as found in faba bean (Easlon and Richards 2009). Recent evidence also indicates this may involve the circadian clock, which is influenced by leaf starch content (Westgeest et al. 2023).

In soybeans, high night temperature decreases stomatal conductance (Shu 2021), possibly linked to reduced starch content (Said et al. 1967; Yang et al. 2023), improving water use efficiency under high night temperature as observed by Shu (2021). This also explains why Cayetano‐Marcial et al. (2021) initially observed slower wilting of common bean under combined drought and high night temperatures compared to drought and ambient temperature.

This contrasts with responses to combined drought and daytime heat stress, where a sharp tradeoff exists between photosynthesis and water conservation. Both isolated drought and combined daytime heat and drought decrease stomatal conductance and photosynthesis, while isolated heat stress increases it (Sehgal et al. 2017). Consequently, combined drought and daytime heat stress typically exceeds the effects of either stress alone (Sehgal et al. 2017), while combined drought and nighttime heat stress may have less severe impacts (Cayetano‐Marcial et al. 2021; Shu 2021).

Despite heat and drought often occurring simultaneously, data on combined stresses in legumes remain limited, largely due to experimental complexity. Available studies suggest variation in tolerance to combined stresses through partial cross‐tolerance mechanisms (Sehgal et al. 2017). All legumes are drought‐sensitive, with yield reductions of 20%–60% at a water deficit of 60%–65% as compared to optimal conditions (Daryanto et al. 2015). Lentils, pea and soybean show greater drought tolerance, while faba bean and chickpea have moderate tolerance, and common bean is highly sensitive (Daryanto et al. 2015).

This has been partially explained by different drought adaptation mechanisms, including stomatal regulation, osmotic adjustment and nodule type as indeterminate nodules, as found in faba bean can recover from drought, while determinant nodules typically die off in similar conditions (Daryanto et al. 2015). As water availability decreases, yields decline in all legumes, though at different rates, depending on species.

Considerable intraspecific variation exists in drought adaptation and yield maintenance under water‐limited conditions. Several traits have been associated with enhanced drought tolerance, including chlorophyll content and photosynthesis rate, root architecture, enhanced photosynthate remobilisation to the grain, proline content, nutrient uptake, water use efficiency and transpiration rate (Chamarthi et al. 2024; Essa et al. 2023; Mansour et al. 2021; Polania et al. 2016). Higher water use efficiency and effective stomatal conductance are particularly important mechanisms in soybean (Minussi Winck et al. 2022; Mutava et al. 2015; X. Wang et al. 2022) and faba bean (Khazaei et al. 2013), for instance.

Several genes and proteins have been identified as associated with heat‐drought‐cross‐tolerance in legumes. These include genes differentially expressed under combined heat and drought stress (Das et al. 2016), genes induced by both heat and drought stress individually, suggesting shared regulatory mechanisms (Kidokoro et al. 2015) and genes associated with general abiotic stress tolerance per se (Panigrahi et al. 2024). Despite these advances in understanding daytime stress responses, comparable data on combined drought and high night temperature stress remain largely unavailable, representing a critical knowledge gap for future research.

#### 3.4.4 Oxidative Stress

During the day, photooxidation represents a major component of heat and drought stress, causing cellular damage through ROS. ROS are generated at photosystems under excess light conditions, a process accelerated under heat or drought (Gururani et al. 2015). As a result, plants with higher antioxidant capacity suffer less damage to photosynthesis and demonstrate greater adaptation to daytime heat stress (Gill and Tuteja 2010).

During the night, the absence of light means photosystems receive no energy input, which substantially reduces ROS production. Indeed, Djanaguiraman et al. (2013) demonstrate that thylakoid damage is indeed more severe under high day temperature stress compared to equivalent high night temperature stress. Nevertheless, high night temperatures still cause thylakoid damage, which is associated with chlorophyll degradation. Albertine and Manning (2009) reported that common bean grown under high night temperature shows increased sensitivity to ozone injury, although their study did not allow a direct comparison to daytime sensitivity.

Interestingly, Djanaguiraman et al. (2013) revealed that NPQ was more strongly induced under high night temperature as compared to high day temperature. These unexpected results are likely a consequence of the differential stacking of thylakoid membranes with more, but smaller grana formed during the night, favouring NPQ and cyclic electron transport to anticipate morning conditions (Wood et al. 2018). These findings suggest that reduced oxidative stress during the night may not only be a result of lower light stress, but also from more favourable grana stacking arrangements. Therefore, when studying oxidative stress responses, researchers should consider the effects of night temperature on thylakoid structure, grana size and the balance between linear and cyclic electron transport pathways.

### 3.5 The Influence of Circadian Clock Genes on Night Temperature Regulation

The circadian clock is a regulatory framework that helps to anticipate regular diurnal changes, including light availability, changes in temperature and even patterns in pathogen activity, as reviewed in Creux and Harmer (2019).

As photoautotrophic organisms, plants exhibit strong circadian influence, with approximately one‐third of all genes directly regulated by the circadian clock (Covington et al. 2008). The clock regulates numerous critical processes, including photosynthesis (Dodd et al. 2014), cold, heat and drought signalling (Kidokoro et al. 2023), flowering time (M. Johansson and Staiger 2015), stomatal opening (Hassidim et al. 2017) and phytohormone responses (Liang et al. 2024). Notably, drought‐tolerant and drought‐sensitive plants show phase‐shifts in their circadian rhythms (Creux and Harmer 2019), indicating the circadian clock's importance in abiotic stress tolerance.

The circadian clock in legumes is largely conserved in *Arabidopsis thaliana* (Kugan et al. 2021), with some small but important distinctions. Most importantly, the role of the GIGANTEA/ZEITLUPE (GI/ZTL) complex functions differently in flowering regulation between long‐day plants like *A. thaliana* and short‐day plants like soybean. Legumes exhibit diverse photoperiod responses. Soybean, common bean, cowpea, mungbean, pigeon pea, groundnut and black gram are short‐day plants, while pea, lentil, chickpea, faba bean and lupin are long‐day plants. These differences likely have implications for developmental interactions with nighttime abiotic stress response, as short‐day plants experience longer nights during flowering, resulting in less oxidative stress and water loss, but also more respiration and less photosynthesis.

The direct involvement of the circadian clock in legume abiotic stress response was established in early research (Gorton et al. 1989; Grzesiak et al. 1989). Gorton et al. (1989) verified circadian rhythmicity of stomatal conductance in functionally isolated guard cells of faba bean leaves, demonstrating that each guard cell pair contains a circadian clock. Under water deficit conditions, the diurnal pattern of stomatal opening in faba bean leaves shifts from two peaks of stomatal opening at 10 AM and 4 PM to a single peak between 8 AM and 11 AM (Grzesiak et al. 1989). Recent research has identified several orthologues of core clock genes involved in stress response in legumes (Table S1).

Legumes present an additional dimension to the influence of the circadian clock on abiotic stress response through Rhizobium symbiosis, which seems to depend on the circadian rhythm of both host plant and symbiont (Rowson et al. 2024). Plants with mutations in central circadian clock genes like *LHY* produce fewer nodules and less biomass under nitrogen‐limited conditions (Rowson et al. 2024). As plants generally experience reduced nutrient uptake under drought, suboptimal nodulation may further compromise their performance under stress conditions.

## 4 Conclusion: Night Temperatures Cannot be Ignored

Nighttime abiotic stress responses in legumes differ significantly from daytime stress responses and warrant particular attention due to rapidly rising night temperatures resulting from climate change. While low night temperatures may lead to lower yield by decreasing biomass and seed size, high night temperatures create metabolic imbalances and affect both pod set and seed size. In this review, we have identified several mechanisms potentially involved in nighttime abiotic stress tolerance in legumes. In particular, balancing nighttime respiration and daytime photosynthesis could help to ensure allocation of resources to reproductive parts and improve pod set. A higher sensitivity to photosynthetic capacity and starch content could also contribute to balancing the effects of combined temperature and drought stress by minimising nighttime water loss. For other mechanisms like membrane lipid oscillation, thylakoid structure and non‐photochemical quenching, as well as direct regulation by the circadian clock, data scarcity is still too critical for final conclusions. We believe that elucidating these mechanisms could be instrumental in identifying genetic variation in nighttime abiotic stress tolerance. This involves the definition of standard protocols for isolated and combined night and daytime temperature stress for each species, as well as screening plant genetic resources to elucidate the amplitude of possible effects and the interplay of daytime and nighttime tolerance mechanisms, although this sometimes includes night phenotyping and sampling. Based on those results, breeders need to define suitable ideotypes with the best achievable combination of independent microtraits, as daytime ROS inactivation and more balanced nighttime respiration, and finally identify genetic markers for marker‐assisted breeding programs. Altogether, this would assist in developing legume cultivars with improved all‐day stress tolerance. As climate change continues to alter temperature patterns worldwide, understanding nighttime temperature responses becomes increasingly critical for sustainable legume production. Our review is meant to stimulate more research towards this important phenomenon to genetically improve yield stability in grain legumes.

## Conflicts of Interest

The authors declare no conflicts of interest.

## Supporting information


**Supplementary Table S1:** List of circadian clock genes potentially involved in temperature response and their occurrence in grain legumes.

## Data Availability

Data sharing is not applicable to this article as no datasets were generated or analysed during the current study.
