# Supplementary material for: The Dark Side of Stress Response: Night Temperature Regimes Drive Distinct Abiotic Pathways in Legumes
Source: Plant Cell Environ. 2025 Nov 3;49(2):925–39. doi: 10.1111/pce.70276 (PMC12779202; doi:10.1111/pce.70276)
Supplement: Supplementary file 1 — Supplementary Table S1: List of circadian clock genes potentially involved in temperature response and their occurrence in grain legumes. [file PCE-49-925-s001.docx]

# Supplementary information

**Supplementary table S1:** List of circadian clock genes potentially involved in temperature response and their occurrence in grain legumes. We list the common gene names as known from *Arabidopsis thaliana*, their role as known from *A. thaliana*, their potential link to temperature response and their occurrence in different grain legumes along with the respective references.

| **Gene names in *A.thaliana*** | **Role in circadian system in *A.thaliana*** | **Temperature response in *A.thaliana*** | **Found in grain legumes** | **References** |
| --- | --- | --- | --- | --- |
| CCA1 and LHY | Repress expression *of PRR9, PRR7, PRR5, TOC1, LUX, ELF4, CCA1, LHY*. | *CCA1* and *LHY* expression is affected by small changes in temperature. The binding of *CCA1* to chromatin is higher at moderately higher temperatures than control temperatures. *CCA1* and *LHY* are more important at lower temperatures. Both extreme heat and cold shocks have been shown to affect the activity of these Myb-like transcription factors. | Soybean | Bian et al., 2017 (https://doi.org/10.3390/ijms18102040 ), Wang et al., 2020 ( https://doi.org/10.1111/nph.17019), Wang et al., 2023 (https://doi.org/10.3390/plants12193344), Michael et al., 2022 (https://doi.org/10.1093/plphys/kiac276), Liu et al., 2009 (https://doi.org/10.1016/j.jplph.2008.06.003), Hudson et al., 2010 ( https://doi.org/10.3835/plantgenome2009.08.0025) |
|  |  |  | Common bean | Kaldis et al., 2003 (https://doi.org/10.1023/A:1025433529082), Yang et al., 2019 (10.21273/JASHS04719-19), Yang et al., 2021(https://doi.org/10.1007/s00438-020-01751-0), Hecht et al., 2007 (https://doi.org/10.1104/pp.104.057018), Liew et al., 2009 (10.1105/tpc.109.067223) |
|  |  |  | Chickpea | Gretsova et al., 2023 (https://doi.org/10.3390/ijms24032692), Kudapa et al., 2023 (https://doi.org/10.3390/ijms24021369) |
|  |  |  | Garden pea | Weller et al., 2009 (https://doi.org/10.1093/jxb/erp120), Hecht et al., 2005 (https://doi.org/10.1104/pp.104.057018), Liew et al., 2009 (10.1105/tpc.109.067223) |
|  |  |  | Faba bean | Aguilar-Benitez et al., 2021 |
|  |  |  | Mung bean | Liu et al., 2022 (https://link.springer.com/article/10.1186/s12864-022-08620-7), Kim et al., 2014 (https://doi.org/10.1007/s13258-014-0215-8) |
|  |  |  | Pigeon pea | Sinha et al., 2020 (https://doi.org/10.1111/pbi.13333) |
|  |  |  | Groundnut | Liu et al., 2023 (https://doi.org/10.1002/adbi.202300410) |
| RVE8/LCL5,  RVE6 and RVE4 | Promote expression of *PRR9, PRR5, TOC1, LUX, ELF4*. | *RVE4, RVE6,* and *RVE8* genes have a temperature-dependent activity, which opposes *CCA1* and *LHY* action. Both extreme heat and cold shocks have been shown to affect the activity of these Myb-like transcription factors. | Soybean | Wang et al., 2023 (https://doi.org/10.3390/plants12193344), Shan et al., 2021 (10.3389/fpls.2021.764074), Michael et al., 2022 (https://doi.org/10.1093/plphys/kiac276) |
|  |  |  | Garden pea | Weller et al., 2009 (https://doi.org/10.1093/jxb/erp120) |
|  |  |  | Pigeon pea | Kumar et al., 2024 (https://doi.org/10.1111/pce.15322), Soni et al., 2024 (10.22541/au.172486704.45525794/v1) |
| TOC1/PRR1 | Represses expression of *CCA1, LHY, RVE8, PRR5,* *TOC1, ELF4, LUX*. | *prr5 toc1* mutants are undercompensated, with a short-period phenotype that is exacerbated at higher temperatures. These proteins undergo enhanced ubiquitination and destabilization at 12°C compared with the optimal growth temperature of 22°C. | Soybean | Wang et al., 2023 (https://doi.org/10.3390/plants12193344), Chen et al., 2015 (https://doi.org/10.1093/jxb/erv407), Liu et al., 2009 (https://doi.org/10.1016/j.jplph.2008.06.003), Hudson et al., 2010 ( https://doi.org/10.3835/plantgenome2009.08.0025), Galeou et al., 2012 (https://doi.org/10.1016/j.plantsci.2011.12.014) |
|  |  |  | Common bean | Yang et al., 2019 (10.21273/JASHS04719-19), Yang et al., 2021(https://doi.org/10.1007/s00438-020-01751-0) |
|  |  |  | Chickpea | Basu et al., 2018 ( https://doi.org/10.1111/pce.13319), Basu et al., 2022 (https://doi.org/10.1007/s11103-022-01247-y) |
|  |  |  | Garden pea | Weller et al., 2009 (https://doi.org/10.1093/jxb/erp120), Hecht et al., 2007 (https://doi.org/10.1104/pp.104.057018), Liew et al., 2009 (10.1105/tpc.109.067223) |
|  |  |  | Mung bean | Yin et al., 2024 (https://doi.org/10.1186/s12870-024-05236-9) |
|  |  |  | Lentil | Hosseini et al., 2022 |
|  |  |  | Faba bean | Aguilar-Benitez et al., 2021 |
|  |  |  | Pigeon pea | Unnikrishan et al., 2024 (https://doi.org/10.1007/s12298-024-01434-9) |
| PRR9, PRR7 and PRR5 | All repress expression of *CCA1, LHY, RVE8. PRR7 and PRR9* directly or indirectly positively regulate expression of *LWD1/LWD2*. *PRR5* regulates *TOC1* nuclear localization. | *PRR7* and *PRR9* are important in temperature entrainment and expression is strongly induced in response to warm pulses given during the night but not the day. *PRR7* transcription is directly repressed by a heat shock factor. *PRR7* and *PRR9* mRNA levels increase rapidly upon transfer of plants from an optimal growth temperature to a slightly stressful condition. *PRR7* and *PRR9* are more important for normal circadian rhythmicity at higher temperatures. | Soybean | Wang et al., 2023 (https://doi.org/10.3390/plants12193344), Michael et al., 2022 (https://doi.org/10.1093/plphys/kiac276) |
|  |  |  | Common Bean | Yang et al., 2019 (10.21273/JASHS04719-19), Yang et al., 2021(https://doi.org/10.1007/s00438-020-01751-0) |
|  |  |  | Garden pea | Weller et al., 2009 (https://doi.org/10.1093/jxb/erp120), Liew et al., 2009 (10.1105/tpc.109.067223) |
|  |  |  | Pigeon pea | Unnikrishan et al., 2024 (https://doi.org/10.1007/s12298-024-01434-9) |
|  |  |  | Groundnut | Khan et al., 2017 (https://doi.org/10.3390/genes8040121) |
| ELF3, ELF4 and LUX/PCL | Transcriptional repressor complex. *LUX* is a Myb-like TF; *ELF4* is a small protein that acts like a ligand to modulate *LUX* activity; *ELF3* acts as a scaffold to assemble the EC. Represses expression of *LUX, GI, LNK1, PRR9, PRR7*. | *ELF3* has a thermosensory function and acts as a temperature-dependent repressor of gene expression, with temperature increases as small as 5°C causing lower affinity binding to target gene promoters, including those of other clock components. Mutation *ELF4* such that it has lower affinity for *ELF3* results in plants with a slightly longer period than wild type, specifically at warm temperatures. *LUX* is found to display significant temperature-dependent differences in splicing, but whether lux mutants have defects in temperature compensation is not determined. | Soybean | Marcolino-Gomez et al., 2017 (10.3389/fpls.2017.00618), Yue et al., 2021 (10.1186/s12864-021-07869-8), Wang et al., 2023 (https://doi.org/10.3390/plants12193344) |
|  |  |  | Common bean | Yang et al., 2019 (10.21273/JASHS04719-19), Yang et al., 2021(https://doi.org/10.1007/s00438-020-01751-0), , Galeou et al., 2012 (https://doi.org/10.1016/j.plantsci.2011.12.014) |
|  |  |  | Chickpea | Basu et al., 2022 (https://doi.org/10.1007/s11103-022-01247-y), Ridge et al., 2017 (https://doi.org/10.1104/pp.17.00082), Perez-Rial et al., 2024 (https://doi.org/10.1186/s12870-024-05411-y) |
|  |  |  | Garden pea | Liew et al., 2009 (10.1105/tpc.109.067223), Liew et al., 2014 (10.1104/pp.114.237008), Rubenach 2017 et al., 2017 (10.1104/pp.16.01738), Weller et al., 2009 (https://doi.org/10.1093/jxb/erp120), Hecht et al., 2005 (https://doi.org/10.1104/pp.104.057018) |
|  |  |  | Mung bean | Ha et al., 2021 (https://doi.org/10.1002/tpg2.20121) |
|  |  |  | Lentil | Roy et al., 2023 |
|  |  |  | Faba bean | Aguilar-Benitez et al., 2021 |
|  |  |  | Pigeon pea | Bhattacharjee et al., 2023 (https://doi.org/10.1007/s10142-023-01236-4) |
| ZTL, FKF1 and LKP2 | Blue-light photoreceptors with light-dependent and light-independent functions in oscillator. *ZTL* and *LKP2* promote degradation of *TOC1* and *PRR5* proteins. *FKF1* degrades transcription factors that inhibit *CO* expression. | *PRR5* and *TOC1* interaction with the *ZTL* homolog *LKP2*, but not *ZTL* itself, is promoted at low temperatures, and *lkp2* but not *ztl* mutants display altered temperature compensation. | Soybean | Li et al., 2023 (10.1111/nph.18826), Li et al., 2013 (10.1371/journal.pone.0079036), Xue et al., 2012 (https://doi.org/10.1007/s11033-011-0875-2) |
|  |  |  | Common bean | Kwak et al., 2008 (https://doi.org/10.1093/jhered/esn005) |
|  |  |  | Chickpea | Gupta et al., 2015 (https://doi.org/10.1186/s12864-015-1293-y) |
|  |  |  | Garden pea | Hecht et al., 2007 (https://doi.org/10.1104/pp.104.057018) |
|  |  |  | Mungbean | Li et al., 2015 (https://doi.org/10.1371/journal.pone.0132969) |
|  |  |  | Pigeon pea | Unnikrishan et al., 2024 (https://doi.org/10.1007/s12298-024-01434-9) |
|  |  |  | Groundnut | Zhang et al., 2016 (https://doi.org/10.1186/s12864-016-2857-1) |
|  |  |  | Adzuki bean | Liu et al., 2016 (https://doi.org/10.1038/srep39523) |
